# Heat-related mortality projections for cardiovascular and respiratory disease under the changing climate in Beijing, China

**DOI:** 10.1038/srep11441

**Published:** 2015-08-06

**Authors:** Tiantian Li, Jie Ban, Radley M. Horton, Daniel A. Bader, Ganlin Huang, Qinghua Sun, Patrick L. Kinney

**Affiliations:** 1 State Key Laboratory of Earth Surface Processes and Resource Ecology, Beijing Normal University, No. 19 Xinjiekouwai Street, Haidian 100875, Beijing, China; 2 Institute for Environmental Health and Related Product Safety, Chinese Center for Disease Control and Prevention, No. 29 Nanwei Road, Xicheng, 100050 Beijing, China; 3 Center for Climate Systems Research, Columbia University, 545 W112th Street, New York, NY 10027 USA; 4 Mailman School of Public Health, Columbia University, 722 West 168th Street, Room 1104E, NY 10032 New York, USA

## Abstract

Because heat-related health effects tend to become more serious at higher temperatures, there is an urgent need to determine the mortality projection of specific heat-sensitive diseases to provide more detailed information regarding the variation of the sensitivity of such diseases. In this study, the specific mortality of cardiovascular and respiratory disease in Beijing was initially projected under five different global-scale General Circulation Models (GCMs) and two Representative Concentration Pathways scenarios (RCPs) in the 2020s, 2050s, and 2080s compared to the 1980s. Multi-model ensembles indicated cardiovascular mortality could increase by an average percentage of 18.4%, 47.8%, and 69.0% in the 2020s, 2050s, and 2080s under RCP 4.5, respectively, and by 16.6%,73.8% and 134% in different decades respectively, under RCP 8.5 compared to the baseline range. The same increasing pattern was also observed in respiratory mortality. The heat-related deaths under the RCP8.5 scenario were found to reach a higher number and to increase more rapidly during the 21^st^ century compared to the RCP4.5 scenario, especially in the 2050s and the 2080s. The projection results show potential trends in cause-specific mortality in the context of climate change, and provide support for public health interventions tailored to specific climate-related future health risks.

With the growing recognition that exposure to extreme heat is associated with increased mortality, heat-related mortality has become a matter of growing public health concern, especially in light of climate change^1^,^2^. Many previous studies focused on the impacts of climate change on future heat-related mortality, including projecting the future mortality change^3^,^4^ by integrating temperature projections from global-scale climate models with historical exposure-response relationships of temperature and mortality from the epidemiologic literature^4^. These studies produced quantitative or qualitative information on future mortality change, which provides decision makers with information regarding heat-related health risk. These studies projected the change of total mortality based on a set of Special Report on Emissions Scenarios (SRES) defined by the Intergovernmental Panel on Climate Change (IPCC)^3^. However, information regarding total mortality change may be less useful for policy decision making than information associated with mortality to specific heat-sensitive diseases, the subject of this manuscript.

As reported in many studies, chronic diseases are significantly sensitive to warm temperature in many cases, especially cardiovascular and respiratory diseases^5^,^6^,^7^,^8^,^9^,^10^,^11^ in many locations of the world. To date, few studies have focused on the impacts of projected climate changes on cause-specific mortality. The existing studies either adopted historical time-series analyses to estimate temperature effects on different diseases^5^ or projected the specific changing mortality trend based on population aging and growth over a short time period^6^, which could not clearly show the changing trend of different diseases caused by projected large temperature increases later this century. Thus there is an urgent need for more research to explore the effect of heat on specific diseases, which could provide direct and specific suggestions for public health intervention policy making, adaption strategy planning, and risk communication. Therefore, we initially attempted to project the specific mortality in China, where few previous studies have examined these issues, while a large population and diverse climate conditions present unique challenges for climate adaptation planning.

In this study, we separately project the mortality change for cardiovascular and respiratory disease in three future 30-year periods in Beijing. We conducted the projections under two Representative Concentration Pathways (RCPs) scenarios: a stabilization scenario (RCP4.5) and an increasing scenario (RCP8.5)^12^. Each RCP refers to a certain amount of ‘climate forcing’ caused by increases in the concentrations of greenhouse gases and other radiatively important agents. This study is an initial exploration in China and is a study unlike most performed around the world. Urban areas are particularly vulnerable to heat because of high concentrations of susceptible people^13^. Beijing is the political and economic center of China, with over ten million people and a rapidly increasing population density. In addition, the process of rapid urbanization and associated frequent occurrence of extreme heat locally^14^ can increase the health risks beyond that associated with climate change. Thus, the projection is extremely important in a metropolitan region such as Beijing, which could expose millions of residents to summer heat stress, resulting in relatively high numbers of additional heat-related deaths. Knowledge of the specific mortality trend could provide detailed information of the relevant health risk. The results of such a projection of the mortality trend can support heat-related health risk management and public health interventions in future decades.

## Methods

We first obtained downscaled future temperature projections for Beijing from five different Global Circulation Models (GCMs) and two of the latest RCP scenarios of greenhouse gas concentrations used in the Intergovernmental Panel on Climate Change Fifth Assessment Report (IPCC AR5). We then cited the exposure-response relationship in Beijing from the study of Liu *et al.*^9^. Finally, we combined the above two sets of information to project the future occurrences of cardiovascular diseases (I00–I99), including acute rheumatic fever, ischemic heart disease, cerebrovascular disease, and other forms of cardiovascular diseases^9^,^15^ and respiratory diseases (J00–J99), which consist of acute upper respiratory infections, influenza and pneumonia, chronic respiratory diseases and other diseases of the respiratory system 15, as well as specified the additional mortality related to future heat effects due to the increased temperature in the future.

### Future Temperature Projections

Future temperature projections were developed using the downscaled outputs from five GCMs from different modeling centers, including ACCESS1.0, CSIRO-Mk3.6.0, GFDL-CM3, GISS-E2-R, and INM-CM4 (see Supplementary Table S1), under two future RCPs scenarios used in the Intergovernmental Panel on Climate Change Fifth Assessment Report (IPCC AR5)^16^,^17^. These GCM simulations are from the Coupled Model Intercomparison Project Phase 5 and were developed for IPCC AR5^18^. The climate model data used here are further downscaled from the bias-corrected and spatially disaggregated (BCSD) dataset, with 0.5 degree resolution^19^,^20^. RCP4.5 is a stabilization scenario in which the total radiative forcing is stabilized shortly after 2100, RCP8.5 is an emissions scenario of high population and high energy demand in absence of climate change policies, which corresponds to the pathway with the highest greenhouse gas emissions^21^. Both of these two scenarios were selected for comparison.

The daily mean temperature output from each model’s single land-based model gridbox covering the center of Beijing was selected. The monthly 30-year means of hind-casts from each climate model over the baseline period from 1971–2000 were subtracted from the 30-year monthly means projected by the same model in the three future periods from 2010–2039, 2040–2069, and 2070–2099 to derive calendar month-specific anomalies. These anomalies were then added to the observed daily temperatures at the Beijing Nanjiao station (39.48 ºN, 116.28 ºE) from 1971–2000 to obtain downscaled climate projections for each GCM/scenario in each of the three future time periods. A set of 10 (5 GCMs × 2 RCPs) future temperature projections for the daily mean temperature from 2010 to 2100 was obtained using the above approach. The three 30-year periods defined are denoted as the 2020s, 2050s, and 2080s, with the 1980s referring to the base period.

### Mortality Risk Projection

The projected heat-related mortality risk was estimated using the modeled daily mean temperature. Because previous studies suggested that the results of projections using different temperature metrics were similar due to the complexity of heat effect impact factors, such as individual health status and exposure^22^, we selected daily mean temperature to present the average situation^7^,^23^,^24^,^25^. We defined the heat effect as a temperature above 21.3 ºC based on the study of Liu et al.^9^, which used different threshold temperatures for a J-shaped function and found that 21.3 °C was the most appropriate threshold temperature based on the Akaike Information Criterion (AIC).

The specific cardiovascular and respiratory mortality were calculated as follows.


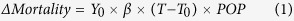


where: 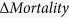 is the daily heat-related additional deaths; *Y*_*0*_ is the baseline daily mortality rate (per 100,000 population); 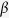
is the exposure mortality relationship for the temperature heat effect; *T* is the projected daily mean temperature; *T*_*0*_is the threshold of the exposure-mortality relationship; *POP* is the population.

The value of 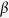 should be obtained from the previous study. However, many exposure-response relationships reported by previous research in Beijing are nonlinear U-, V- or J- shape, which are not appropriate for projection. Liu’s study in Beijing quantified cold and heat effects separately and assumed a linear response above a threshold temperature, making the projection available^3^,^23^,^26^. According to the studies of Liu *et al.*, above the local threshold temperature of 21.3 ºC, the relative risk of daily cardiovascular disease (I00–I99) mortality and daily respiratory disease (J00–J99) mortality in association with a 5 ºC increase of the 2-day average temperature above the threshold for the heat effect is 1.098 (95% confidence interval (95%CI):1.057–1.140) and 1.134 (95%CI: 1.050–1.224), respectively. The population of Beijing was 1,257,8000, based on the data obtained from the China census 2010 survey, and was held constant throughout the projection period. The baseline cardiovascular mortality was 0.396 per 100,000 populations, and the baseline respiratory mortality was 0.085 per 100,000 populations for all ages obtained from the study of Liu *et al.*^9^, which was also held constant in our projection.

## Results

**Figure 1:**
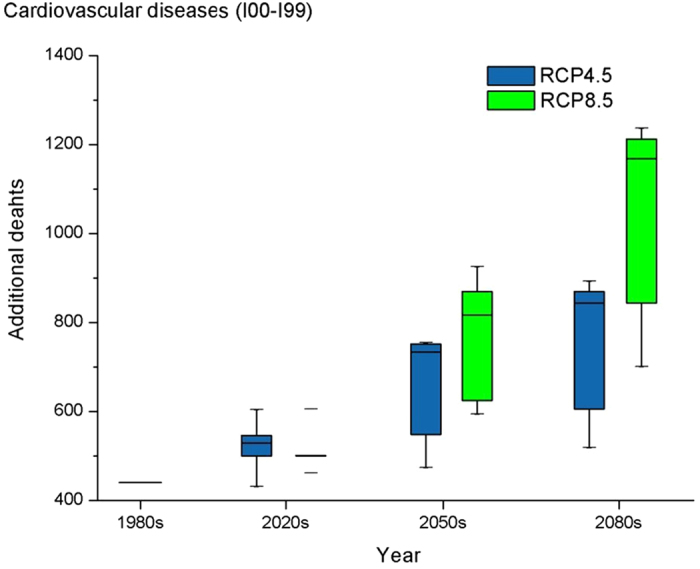
Distribution of heat-related additional deaths for cardiovascular diseases in the 1980s, 2020s, 2050, and 2080s for 5 climate models and for the RCP4.5 and RCP8.5 future climate scenarios. The box symbols represent, from bottom to top, the minimum, 25th percentile, 50^th^ percentile, 75^th^ percentile and maximum across the 5 models.

**Figure 2:**
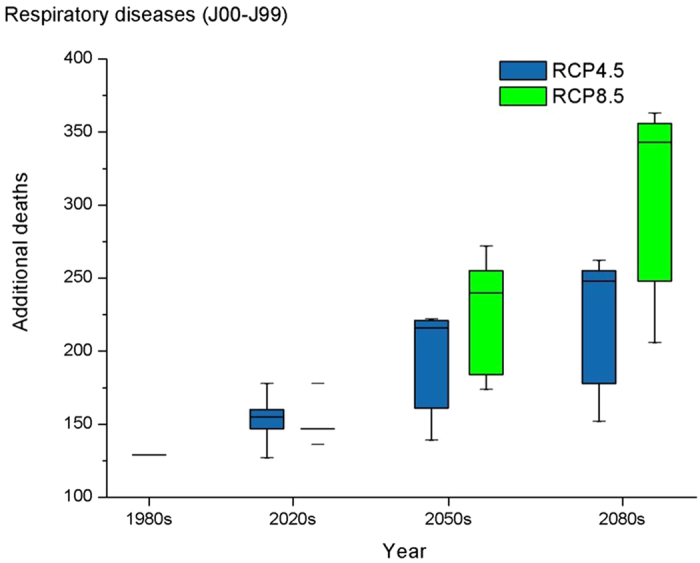
Distribution of heat-related additional deaths for respiratory diseases in the 1980s , 2020s, 2050, and 2080s for 5 climate models and for the RCP4.5 and RCP8.5 future climate scenarios. The box symbols represent, from bottom to top, the minimum, 25th percentile, 50^th^ percentile, 75^th^ percentile and maximum across the 5 models.

Figures 1 and 2 graphically summarize the projected absolute changes in heat-related additional deaths from the 1980s to the 2080s. The results are presented separately for cardiovascular and respiratory deaths under different scenarios. For all 5 GCMs and both RCP climate scenarios, additional heat-related deaths increase compared to the baseline as time progresses. The median numbers of additional cardiovascular deaths in the 2020s, 2050s and 2080s under RCP 4.5 were 529, 734 and 844, respectively, and the increasing percentage ranged across GCMs from −2% to 37%, 7% to 71% and 18% to 102% in the2020s, 2050s and 2080s, respectively, with a mean increase of 18.4%, 47.8%, 69.0% in the 2020s, 2050s and 2080s, respectively, compared with the 1980s. The median numbers of additional cardiovascular deaths under RCP 8.5 were higher, with values of 501, 817, 1168 in the 2020s, 2050s and 2080s, respectively, with a mean increase of 16.6%, 73.8% and 134% in the 2020s, 2050s and 2080s, respectively, compared to the baseline year. The same increasing pattern was seen in respiratory disease under the two scenarios: in the RCP4.5 scenario, there were 155, 216 and 248 additional deaths in the 2020s, 2050s and 2080s, respectively, with an average increasing percentage of 18.80%, 48.60% and 69.80% in the 2020s, 2050s and 2080s. Under RCP 8.5, the increases were 17.0%, 74.6% and 135% in the 2020s, 2050s and 2080s, respectively. Therefore, larger increases were seen in the RCP8.5 scenario, especially in the 2050s and the 2080s. The heat-related deaths under the RCP8.5 scenario increased more rapidly from the 1980s to the 2080s compared to the RCP4.5 scenario. This situation was seen in both cardiovascular and respiratory disease. The detailed information is presented in Supplementary Table S2.

**Figure 3:**
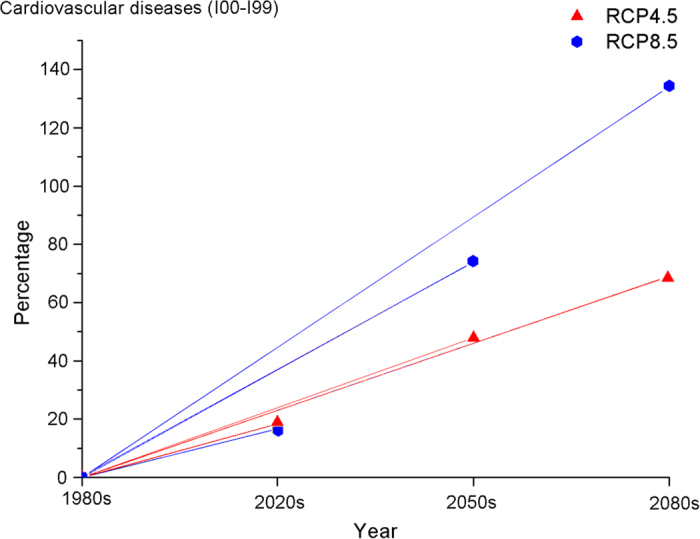
Percentage change (average over 5 models) in heat-related deaths from cardiovascular diseases in the 2020s, 2050s, and 2080s versus the 1980s under the RCP4.5 and RCP8.5 future climate scenarios.

**Figure 4:**
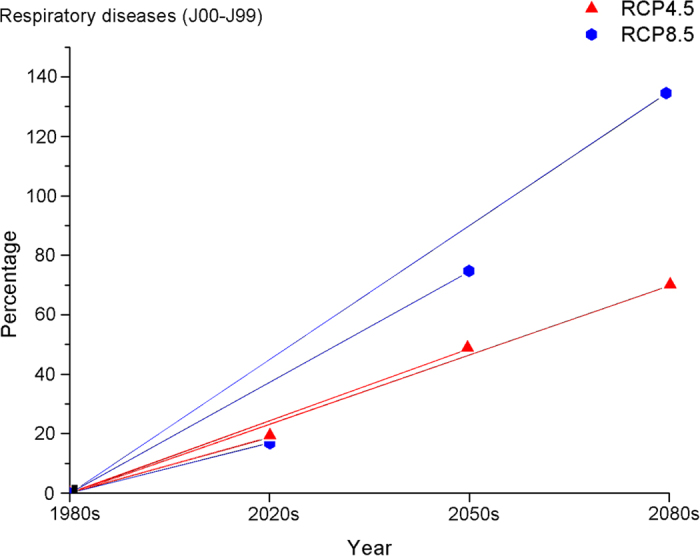
Percentage change (average over 5 models) in heat-related deaths from respiratory diseases in the 2020s, 2050s, and 2080s versus the 1980s under the RCP4.5 and RCP8.5 future climate scenarios.

Figures 3 and 4 report the percentage changes in the estimated heat-related deaths from two diseases in two scenarios for the 2020s, 2050s and 2080s compared to baseline mortality. A similar pattern of change in additional deaths was also observed in the percentage change. The integrated results of the five GCMs indicated large increases for both diseases under different scenarios across all of the decades. The percentage in the RCP8.5 scenario increases more sharply than in the RCP4.5 scenario after the 2020s and becomes more obviously different in the 2050s and the 2080s. The projected changes for the 2050s in the heat-related mortality of cardiovascular diseases of different scenarios range from an increase of 47.8% to an increase of 73.8% compared with the 1980 s; the 2080 s change ranged from an increase of 69.0% to an increase of 134% compared with the 1980 s. Similar to the changes in the cardiovascular diseases, the projected 2050 s changes in heat-related mortality from respiratory diseases in different scenarios ranged from an increase of 48.6% to an increase of 74.6% compared with the 1980s; the 2080s changes range from an increase of 69.8% to an increase of 135% compared with the 1980s. These results indicate the potential large effect climate change could have on future heat-related mortality.

## Discussion

This study projected additional deaths from both cardiovascular and respiratory diseases in China under changing environmental temperature scenarios according to five different GCMs and two RCPs. The projection was designed differently from previous studies. Firstly, we attempted to explore the changes of specific diseases instead of projecting the total heat-related mortality, resulting in estimates of additional death numbers for both cardiovascular and respiratory diseases. Secondly, we applied the two of the latest RCP climate scenarios addressed by the IPCC to replace the SRES scenarios so that we could project the future temperature change with different technological, socio-economic and policy aspects, while accounting for the current studies. Because climate scenarios have been used as a key approach for policy making and planning in the context of uncertain future conditions, both the current and future heat-related mortality projections should apply the latest RCP scenarios rather than the previous SRES-based projections. The RCP scenarios are comprised of a comprehensive data set with high spatial and sectoral resolutions for the period extending to year 2100, which integrates demographic, technological, political, social and economic development, emissions mitigation and impact analyses^12^. So far, health sector projection studies based on RCPs have been very limited. In addition, we incorporated temperature projections from 5 state-of-the-art GCMs under the two RCP scenarios to obtain a range of changing mortality based on the different regional temperature sensitivity of each model to greenhouse gas concentrations, and to sample the true uncertainty, which could only be weakly estimated using a single GCM or an ensemble average across GCMs. Three 30-year future time periods were selected, the 2020s, 2050s and 2080s, compared to the baseline period. We chose the 1980 s as our modeling baseline because this decade is at the center of the conventional climatological baseline period from 1971–2000. Finally, we assumed no demographic change for our calculations of temperature-related mortality; changes in other factors that influence population vulnerability, such as general health, access to health care, and access to public health messaging, were also assumed to be stable in the future decades^4^. These possible effects may be considered in future studies.

Because previous studies found that the heat effects on cardiovascular and respiratory diseases are obvious^1^,^9^, these two diseases should be of greater concern. These studies have reported different exposure-response relationships between temperature and cardiovascular disease as well as respiratory disease, although there are many more studies reporting cardiovascular disease than those reporting respiratory disease^15^,^27^. Liu’s study found that the exposure-response coefficient is 9.8% per 5 °C for cardiovascular disease and 13.4% per 5 °C for respiratory disease; Braga’s study^5^ indicated that a 1 °C increase is associated with 1%−6% increase in cardiovascular mortality; in another study, respiratory mortality increased by 18.7% when the temperature increased 1 °C^28^. The different case studies all suggested a significant heat-effect on both cardiovascular and respiratory mortality. In this study, the future projection of the mortality of cardiovascular disease was significantly higher than that of respiratory disease due to the different baseline mortality rate. However, a similar pattern of mortality change for these two diseases was also observed. The impacts of the projected rising temperature on heat-related mortality of both diseases may increase over future decades. The mean increasing percentage for both diseases was very close, ranging from 18%–70% in the RCP4.5 scenario and 17%–135% in the RCP8.5 scenario. In addition, for each disease, the estimated temperature warming impacts on additional mortality under different scenarios were similar in the 2020 s but began to diverge sharply in the 2050 s and then differed substantially by the 2080 s. The larger increase of mortality was noted in the increasing scenario (RCP8.5) than the stabilization scenario (RCP4.5), which was more obvious past the 2050 s. Thus, adults with cardiovascular and respiratory diseases could be at greater risk in a warmer climate, thereby justifying public interventions addressing climate change.

The increasing trend found in this study is consistent with those of previous studies that have applied climate model projections in mortality assessments. Many studies projected that the excess total mortality ranged from 45% to 60% by the 2050s^23^,^29^ and 60–70% by the 2080s^29^. Additionally, the mortality further increased in the high emission scenarios, due to its higher temperatures, than the low emission scenarios^30^. Although there is little direct comparability between the results of these projections and ours due to the difference of the mortality pattern, scenarios, study period, locations, and other projecting conditions, we still observed a similar trend of increasing mortality caused by rising temperatures in the future. Some studies indicated that people with cardiovascular or respiratory disease are especially at greater risk from heat exposure^1^,^7^. A recent study on health transition in China reported that the number of deaths and age-standardised death rate from cardiovascular diseases in 2010 were 3,136.2 thousands (2,827.1–3,274.6) and 230.8 per 100,000 (207.4–241.1), becoming the leading causes of disability adjusted life years (DALYs) in 2010; chronic respiratory diseases deaths number and age-standardised death rate were 1,022.9 thousands (965–1,089.3) and 77.2 per 100,000 (72.8–82.3) in 2010. This showed that mortality rates from cardiovascular and respiratory diseases were significantly higher than the rates associated with other diseases^15^. According to our study, the mortality of these two diseases may increase with rapid temperature rise under climate change in future: the results indicated that the annual additional deaths from cardiovascular disease in Beijing could increase by 18.4%-69.0% in the stabilization scenario and by 16.6%–134% in the increasing scenario. Similar to the observations for cardiovascular disease, the additional deaths from respiratory disease increased by 18.8%-69.8% in the stabilization scenario and by 17.0%–135% in the increasing scenario. Therefore, continued attention to these diseases control is required and consistent epidemic surveillance is a necessity^15^.

Few studies focusing on specific cause-of-death projections under climate change are found throughout the environmental health field. Martens reported estimates of the average mortality change of cardiovascular disease vary from −15% to 2% and that of respiratory disease vary from −4% to 25% in 20 global cities according to 3 GCMs from 2040–2100^7^. Yang’s study reported that cardiovascular disease increased 30.6% in China between 1990 and 2010, indicating rapid disease transition due to rapid environmental changes^15^. Based on this conclusion, our study has demonstrated that temperature variations may be an important aspect among rapid environmental change factors, which may lead to mortality change for sensitive diseases in future. However, these studies only reported the change of cardiovascular and respiratory mortality in one future period; as a result, we could not compare the mortality in different future time periods, that is, the long-term changing trend of specific mortality could not be clearly presented. Out study is an initial exploration of future trends in temperature-sensitive diseases under climate change. Cross-study comparisons are needed; however, studies of specific cause-of-death projections under climate change in China are even harder to find. In addition, because of the different types of scenarios, the models, the locations and the time periods applied, we cannot compare the results to the previous studies directly.

Environmental temperatures place extra strain on the cardiovascular and respiratory system^1^. People with cardiovascular or respiratory disease are at greater risk from heat exposure. In the future, if the temperature continues to rise, based on our study, a stronger impact on cardiovascular and respiratory diseases will lead to larger additional mortality, unless adaptive strategies are advanced. Thus, adaptation policies and intervention strategies to address rising temperatures are required to reduce the heat impacts of specific diseases. Climate-health intervention strategies could help at-risk populations to adapt to climate change because climate change is associated with people suffering from cardiovascular and respiratory diseases and with high-exposure populations^31^. In the future, an integrated health risk assessment may eventually help local planners strengthen climate impact adaptations, such as increasing the application of air conditioning, heat alerts and cooling shelters; in addition, gradual physiological adaptation could ameliorate significantly the exposure to heat stress^4^,^23^.

This study projected the heat effect on specific diseases based on multiple models and scenarios and identified cardiovascular and respiratory diseases as a category that requires extra care in hot weather. The results indicate that the health risk posed to exposed populations is projected to increase with temperature in future decades. This disease-specific exploration is meaningful for public health intervention because it could inform disease-specific adaptations. Future work will apply more climate models and more specific diseases, in order to provide a more refined picture of dynamic changes in diseases of concern. More research is also needed to upscale these projections for Beijing to larger spatial scales and for other cities. Population growth and aging may pose higher heat-related mortality risk in the future. Baseline and projections research is also needed on air quality interactions with temperature, as well as joint impacts of air quality and temperature on cardiovascular and respiratory health.

## Additional Information

**How to cite this article**: Li, T. *et al.* Heat-related mortality projections for cardiovascular and respiratory disease under the changing climate in Beijing, China. *Sci. Rep.* **5**, 11441; doi: 10.1038/srep11441 (2015).

## Supplementary Material

Supplementary Information
