# Supplementary material for: Heat-related mortality projections for cardiovascular and respiratory disease under the changing climate in Beijing, China
Source: Sci Rep. 2015 Aug 6;5:11441. doi: 10.1038/srep11441 (PMC4527092; doi:10.1038/srep11441)
Supplement: Supplementary Information [file srep11441-s1.doc]

**Heat-related mortality projections for cardiovascular and respiratory disease under the changing climate in Beijing, China**

**Author list**

Tiantian Li^1, 2^*, Jie Ban^2^, Radley M. Horton^3^, Daniel A. Bader^3^, Ganlin Huang^1^, Qinghua Sun^2^, Patrick L. Kinney^4^*

1. State Key Laboratory of Earth Surface Processes and Resource Ecology, Beijing Normal University, Beijing, China

No. 19 Xinjiekouwai Street, Haidian District, Beijing, 100875 China

1. Institute for Environmental Health and Related Product Safety, Chinese Center for Disease Control and Prevention, Beijing, China

No. 29 Nanwei Road, Xicheng District, Beijing, 100050 China

1. Center for Climate Systems Research, Columbia University, New York, USA

545 W112th Street, New York, NY 10027 USA

1. Mailman School of Public Health, Columbia University, New York, USA

722 West 168th Street, Room 1104E, New York, NY 10032 USA

Tiantian Li

E-mail: [tiantianli@gmail.com](mailto:tiantianli@gmail.com) Tel: 8610-83132372 Fax: 8610-83132372

Jie Ban

E-mail: [bjking99@126.com](mailto:tiantianli@gmail.com)

Radley M. Horton

E-mail: [rh142@columbia.edu](mailto:rh142@columbia.edu)

Daniel A. Bader

E-mail: [dab2145@columbia.edu](mailto:dab2145@columbia.edu)

Ganlin Huang

 E-mail: [ghuang@bnu.edu.cn](mailto:ghuang@bnu.edu.cn)

Qinghua Sun

E-mail: [qhsunpku@gmail.com](mailto:qhsunpku@gmail.com)

Patrick L. Kinney*

E-mail:plk3@columbia.edu Tel: 8610-2123053663 Fax: 8610-2123054012

***To whom correspondence should be addressed.**

| **Table S1** List of the climate models | | |
| --- | --- | --- |
| Model | Modeling center | Institution |
| ACCESS1.0 | CSIRO-BOM | CSIRO (Commonwealth Scientific and Industrial Research Organization, Australia), and BOM (Bureau of Meteorology, Australia) |
| CSIRO-Mk3.6.0 | CSIRO-QCCCE | Commonwealth Scientific and Industrial Research Organization in collaboration with the Queensland Climate Change Centre of Excellence, Australia |
| GFDL-CM3 | NOAA GFDL | Geophysical Fluid Dynamics Laboratory, USA |
| GISS-E2-R | NASA GISS | NASA Goddard Institute for Space Studies, USA |
| INM-CM4 | INM | Institute for Numerical Mathematics, Russia |

In all cases, heat-related additional annual deaths increased in future decades. Under the RCP4.5 scenario, heat-related deaths of cardiovascular diseases increased on average by 0.18% (range across models: -2%-37%) in the 2020s, 48% (7%-71%) in the 2050s, and 69% (18%-102%) in the 2080s, all compared with a climate baseline in the 1980s. The annual deaths of respiratory diseases increased on average by 0.19% (range across models: -2%-38%) in the 2020s, 49% (8%-72%) in the 2050s, and 70% (18%-103%) in the 2080s, all compared with a climate baseline in the 1980s.

Larger increases were seen for the RCP8.5 scenario, especially in the 2050s and 2080s. Heat-related additional mortality projections from the GFDL-CM3 model increased most sharply from the 1980s to 2080s among all 5 models under both scenarios. Increases projected by the INM-CM4 model were lowest.

| **Table S2** Percentage change in annual heat-related deaths in the 2020s, 2050s, 2080s as compared with the baseline year*. | | | | | | | |
| --- | --- | --- | --- | --- | --- | --- | --- |
| Scenario | GCMs | Cardiovascular diseases^*^ | | | Respiratory diseases^#^ | | |
|  |  | 2020s | 2050s | 2080s | 2020s | 2050s | 2080s |
| RCP4.5 | ACCESS1.0 | 0.24 | 0.66 | 0.91 | 0.24 | 0.67 | 0.92 |
|  | CSIRO-Mk3.6.0 | 0.13 | 0.71 | 1.02 | 0.14 | 0.71 | 1.03 |
|  | GFDL-CM3 | 0.37 | 0.71 | 0.97 | 0.38 | 0.72 | 0.98 |
|  | GISS-E2-R | 0.20 | 0.24 | 0.37 | 0.20 | 0.25 | 0.38 |
|  | INM-CM4 | -0.02 | 0.07 | 0.18 | -0.02 | 0.08 | 0.18 |
|  | Mean | 0.18 | 0.48 | 0.69 | 0.19 | 0.49 | 0.70 |
| RCP8.5 | ACCESS1.0 | 0.14 | 0.97 | 1.75 | 0.14 | 0.98 | 1.76 |
|  | CSIRO-Mk3.6.0 | 0.13 | 0.85 | 1.65 | 0.14 | 0.86 | 1.66 |
|  | GFDL-CM3 | 0.37 | 1.1 | 1.8 | 0.38 | 1.11 | 1.81 |
|  | GISS-E2-R | 0.14 | 0.42 | 0.91 | 0.14 | 0.43 | 0.92 |
|  | INM-CM4 | 0.05 | 0.35 | 0.59 | 0.05 | 0.35 | 0.6 |
|  | Mean | 0.17 | 0.74 | 1.34 | 0.17 | 0.75 | 1.35 |
| *Percentage changes relative to 1980s cardiovascular disease annual heat-related deaths of 441;  # Percentage changes relative to 1980s respiratory disease annual heat-related deaths of 129. | | | | | | | |
